# Fissure of the Rectum

**Published:** 1878-02-20

**Authors:** Erskine Mason

**Affiliations:** Professor of Clinical Surgery, in the Bellevue Hospital Medical College


### FISSURE OF THE RECTUM.

### CLINICAL REMARKS MADE AT BELLEVUE HOSPITAL.

BY ERSKINE MASON, M.D.,
Professor of Clinical Surgery, in the Bellevue Hospital Medical College.

Reported for the MEDICAL RECORD.]

GENTLEMEN—The patient now before you is suffering from an affection which in itself is very slight, but one that is sometimes attended by most distressing symptoms—she has fissure of the rectum.

Patients suffering from the disease usually complain of a severe burning pain, occurring most commonly a few minutes after having a movement from the bowels, and especially if the fæces are at all hardened. This pain may last for hours, and give the patient very great distress. It is probably the single symptom which is most characteristic of the affection, and is sufficient to warrant a most careful search for fissure of the rectum.

A common cause of this trouble is constipation. Under such circumstances, when an evacuation from the bowels does occur, it may be large, the hardened mass perhaps tears the mucous membrane, and a fissure is established. On the other hand, fissure of the rectum may follow an attack of diarrhœa. Again, the fissures sometimes result from specific disease.

They may be situated directly opposite the coccyx, or towards the perineal region, or upon either side of the rectum.

It is not unfrequently the case that patients suffer from this affection a long time before they seek relief. They suppose that they are suffering from piles, and when they come to you for advice and describe their case, unless you are upon your guard, it may be mistaken for one of hemorrhoids, and a useless recommendation made. It frequently happens with fissure of the rectum that more or less of blood is lost with each movement of the bowels. That fact alone may lead you astray, and bring you to the conclusion that the case is one of bleeding piles. Again, when you make an examination, you will frequently find the integument along the side of the fissure inflamed and œdematous, and a superficial investigation may lead you to the conclusion that the patient is suffering from an inflamed external hemorrhoid. The only manner, however, in which you will be able to make a correct diagnosis, is by a careful and complete examination.

As you examine these cases, you will almost invariably find associated with the fissure a little papillary enlargement which may have the appearance of a polypus. This polypoid appearance is due to simple hypertrophy of one or two papillæ in the neighborhood of the fissure. These enlarged papillæ may overlap the fissure, if it be a small one, and so conceal it from view. It is important, therefore, in your examination, if such a growth is present, to turn it to one side, and then may be exposed a slight fissure or crack, or grayish line in the mucous membrane that had previously escaped notice. This affection has also received the name of ulcer of the rectum.

The diagnosis having been made, the question arises, how shall the fissure be treated? Can it be treated successfully without resorting to operative procedure?

TREATMENT WITHOUT OPERATION.

In young subjects, and where the fissure is of recent origin, you can, in many cases, succeed in curing them without an operation. The treatment is to keep the bowels in a soluble condition, and make use of some astringent and sedative application. A very common prescription of this kind contains zinc or stramonium ointment in combination with belladonna or opium. This plan of treatment is often followed by complete relief.

There are many persons who are remarkably timid when anything like operative interference is suggested, and you will be able to relieve a goodly number of such cases by penciling the fissure to its bottom with a fine point of nitrate of silver, or with nitric acid. These applications relieve the pain, because they destroy the little filament of nerve which is exposed in the fissure.

In those cases in which the fissure has attained some size, you can always with the probe find one spot which is excessively tender, and when the nerve exposed at that point is destroyed by the use of any cautery, or by stretching the sphincter, the patient will be relieved.

RADICAL TREATMENT.

What is the radical treatment of this painful affection? It consists in dividing the mucous membrane and some of the fibres of the sphincter muscle with a knife. The object is to divide the filament of nerve involved, and, at the same time to put the parts in a complete state of rest. If the nerve-filament is left exposed, all the efforts made by nature towards effecting a cure are frustrated, because every time it is touched by the passage of fæcal matter of the sphincter it is thrown into spasmodic action, and the reparative process is destroyed. If the nerve is destroyed, and the parts put to sleep, as it were, the fissure is quickly cured. This can be accomplished in two ways. First, by over-distention of the rectum. Introduce your thumbs, back to back, into the rectum, make forcible distention towards the tuber ischii, and carry it to the fullest extent possible. In that manner you will completely paralyze the sphincter, if the distension is done thoroughly, and perfect recovery usually follows. There is some danger, however, attending this operation, for you may lacerate the mucous membrane, or you may rupture some vein of considerable size which may give rise to troublesome hemorrhage. Such accidents are not common, but they may occur. The second way in which the nerve filament in the fisssure can be destroyed and the parts put at rest, is by means of a cutting operation. In performing this you should always remove the little enlargement of papillæ which so commonly is present by the side of the fissure, for if you do not, the chances are that your operation will be a failure. The operation itself is very simple, and consists in this. Put the parts upon a stretch by introducing a speculum; the speculum is to be preferred to the fingers. When the parts have been moderately tense, simply draw your knife through the base of the fissure, and divide a few of the fibres of the sphincter muscle; it is not necessary to divide the muscle completely, but simply a few fibres. In this manner you divide the ulcerated mucous membrane, the irritated filament of nerve, and more or less of the fibres of the sphincter below, so that the parts are placed at rest. All that is necessary in the way of after-treatment is, to keep the patient in bed a day or two, and keep the bowels quiet. Before permitting the bowels to move, it is well to give an enema of sweet oil, or some gentle laxative.

#### FISTULA AND FISSURE.

You will recollect I told you that in some cases the integument by the side of the fissure would be inflamed and œdematous, and perhaps to such an extent as to give it the appearance of an inflamed external hemorrhoid. This irritation and inflammation may go on until an abcess is developed. Such an abscess will burst, and when you make an examination it may be found that there is a small fistula formed. The point to which I wish to direct your attention is this: if you are not thorough in your examination, you may operate on the fistula only, and the patient will probably be cured of it, but the same agonizing pain will continue to be experienced after every evacuation from the bowels, simply because the fissures are present, and the question will arise—how are they to be managed? It is not necessary to divide them all, for if one is thoroughly divided, the sphincter will be placed at rest, and the others will heal very readily..

